# preAssemble: a tool for automatic sequencer trace data processing

**DOI:** 10.1186/1471-2105-7-22

**Published:** 2006-01-17

**Authors:** Alexei A Adzhubei, Jon K Laerdahl, Anna V Vlasova

**Affiliations:** 1 Norwegian School of Veterinary Science, BasAM – Genetics, P.O. Box 8146 Dep, NO-0033 Oslo, Norway; 2 Centre for Molecular Biology and Neuroscience (CMBN) Institute of Medical Microbiology, Rikshospitalet, NO-0027 Oslo, Norway; 3 Engelhardt Institute of Molecular Biology, Vavilov St. 32, 117984 Moscow, Russia; 4 The Biotechnology Centre of Oslo, University of Oslo, P.O. Box 1125 Blindern, NO-0317 Oslo, Norway

## Abstract

**Background:**

Trace or chromatogram files (raw data) are produced by automatic nucleic acid sequencing equipment or sequencers. Each file contains information which can be interpreted by specialised software to reveal the sequence (base calling). This is done by the sequencer proprietary software or publicly available programs. Depending on the size of a sequencing project the number of trace files can vary from just a few to thousands of files. Sequencing quality assessment on various criteria is important at the stage preceding clustering and contig assembly. Two major publicly available packages – Phred and Staden are used by preAssemble to perform sequence quality processing.

**Results:**

The preAssemble pre-assembly sequence processing pipeline has been developed for small to large scale automatic processing of DNA sequencer chromatogram (trace) data. The Staden Package Pregap4 module and base-calling program Phred are utilized in the pipeline, which produces detailed and self-explanatory output that can be displayed with a web browser. preAssemble can be used successfully with very little previous experience, however options for parameter tuning are provided for advanced users. preAssemble runs under UNIX and LINUX operating systems. It is available for downloading and will run as stand-alone software. It can also be accessed on the Norwegian Salmon Genome Project web site where preAssemble jobs can be run on the project server.

**Conclusion:**

preAssemble is a tool allowing to perform quality assessment of sequences generated by automatic sequencing equipment. preAssemble is flexible since both interactive jobs on the preAssemble server and the stand alone downloadable version are available.

Virtually no previous experience is necessary to run a default preAssemble job, on the other hand options for parameter tuning are provided. Consequently preAssemble can be used as efficiently for just several trace files as for large scale sequence processing.

## Background

The preAssemble pipeline is intended to be used in sequencing projects at the stage immediately following sequencing and preceding sequence clustering and contigs assembly. Sequence data is produced by automatic sequencers in the form of trace files or chromatograms. Each file normally contains one sequence. Sequencer files are encoded according to the proprietary format used by the sequencing equipment; the formats vary for different equipment manufacturers. The files usually hold both the chromatogram, i.e. raw data and the sequence which is produced (base called) by the software used in the sequencer. Several publicly available packages can be used to perform base calling and quality assessment. One of the most widely used programs is Phred [1,2]. Phred is routinely employed as part of the software used in automatic sequencers. Staden package [3,4] can be used for the whole cycle of sequence data processing, clustering and contig assembly. Phred can be run in conjunction with the Staden package. The purpose of developing preAssemble was to generate reproducible and easily interpreted assessments of the quality of sequencing data by utilising standard software packages such as Phred and Staden with a set of parameters applied consistently to all processed data, independently of its source. Base calling and the initial sequence quality assignment is done by Phred at the initial stage of data processing. Subsequent steps of data processing are performed with the Staden package. Submitting a job on the preAssemble server does not require prior knowledge of Phred or Staden, and can be used for quality testing of sample chromatograms and small scale sequence processing. A stand alone version of preAssemble can be downloaded, installed and used for production large scale data processing.

## Implementation

### Accessing preAssemble on the SGP web server

The preAssemble pipeline is accessible on the Norwegian Salmon Genome Project (SGP) web site [5] from the preAssemble page. Users can upload their trace files and run preAssemble on the SGP server interactively or receive results notification by email. Results can be downloaded as a compressed (zip) archive. This option can be used for small data sets typically not larger than 300 – 400 traces. There is an option on the preAssemble web page allowing to request an update of the vector – primer data if the required parameters are not present in the current selection menu. When computationally intensive processing is necessary users are advised to download and install a local copy of preAssemble from the SGP web site.

### Pipeline operation

preAssemble utilizes Phred and Staden Package Pregap4 to process trace files (chromatograms). Phred can recognise a number of sequencing machine and chemistry combinations and convert into the SCF format used by Pregap4. Thus it is possible to include trace files created by different machines into a single dataset submitted to preAssemble. Pregap4 performs several processing steps listed below. For Pregap4 to work, on a preAssemble web server page the user must select from a drop down menu a single vector-primer combination for the whole submitted dataset. This limitation is due to the Staden package mode of operation which cannot be changed in this pipeline. We suggest that before submitting to the preAssemble web server the data is divided into subsets according to their vector-primer pairs. This will also create more compact datasets which are easier to upload to the server. Processing data in batches of smaller size will increase efficiency since depending on the network speed a large proportion of the online processing time can be taken up by uploading a large data file to the server.

The stand-alone version of the pipeline is run from UNIX/LINUX command line. The Staden Pregap4 parameter file provided with preAssemble contains default settings; the vector-primer subset is specified as a command line parameter. Settings can be changed by users wishing to specify their own parameters.

We would like to note that the Phred and Staden packages are not part of the preAssemble distribution and should be obtained separately. The information on how to obtain this software is provided in the Help file distributed with preAssemble.

The pipeline performs the following main operations.

(1) Source data format conversion. Software used: Phred. Trace files are converted from ABI, ESD and other formats to the universal SCF file format. This is done to ensure full format compatibility between Phred and Staden Package, i.e. that all traces with formats recognized by the current version of Phred can be used with the Staden modules.

(2) Base-calling and assignment of confidence values. Software used: Staden Pregap4/Phred. Base-calling is performed with Phred, which also assigns quality (confidence) values. These values are written to the EXP files.

(3) Quality and vector clip. Software used: Staden Pregap4. Each sequence is checked for low quality regions by reading Phred quality values and calculating the average confidence level for a sliding window. Fragments of sequencing vectors if present are identified by comparison with the vector data set provided as part of the Staden package. Low quality and vector regions are marked in the EXP files for each sequence.

(4) Screening for unclipped vector, poly-A clip and sequence screening for *E.Coli *contamination. Software used: Staden Pregap4. At the final stage of processing sequences are checked for any undetected matches with sequencing vector and for contamination with *E.Coli *sequences. If a match is found the sequence is rejected. Theses two procedures provide a final check-up search in the identified "good" insert sequence for matches with the sequencing vector, and with *E.Coli *sequences. If found such matches almost always show that either no insert had been reliably identified anywhere in the sequence or the insert sequence is in fact an *E.Coli *sequence. In both cases such sequence contaminates the dataset without adding any value to its data; therefore it is removed from the dataset. The 5' and 3' ends of sequences are also checked for occurrences of poly-A and poly-T which if detected are marked in the EXP file. If the final length of 'clipped' good quality sequence is less than a minimum length (preAssemble default 100 bases) it is rejected.

(5) Output data processing. At the post-processing stage a number of output files and directories are created. The output is processed to create the html results files, which can be viewed with a web browser.

## Results and discussion

### preAssemble output

**Figure 1:**
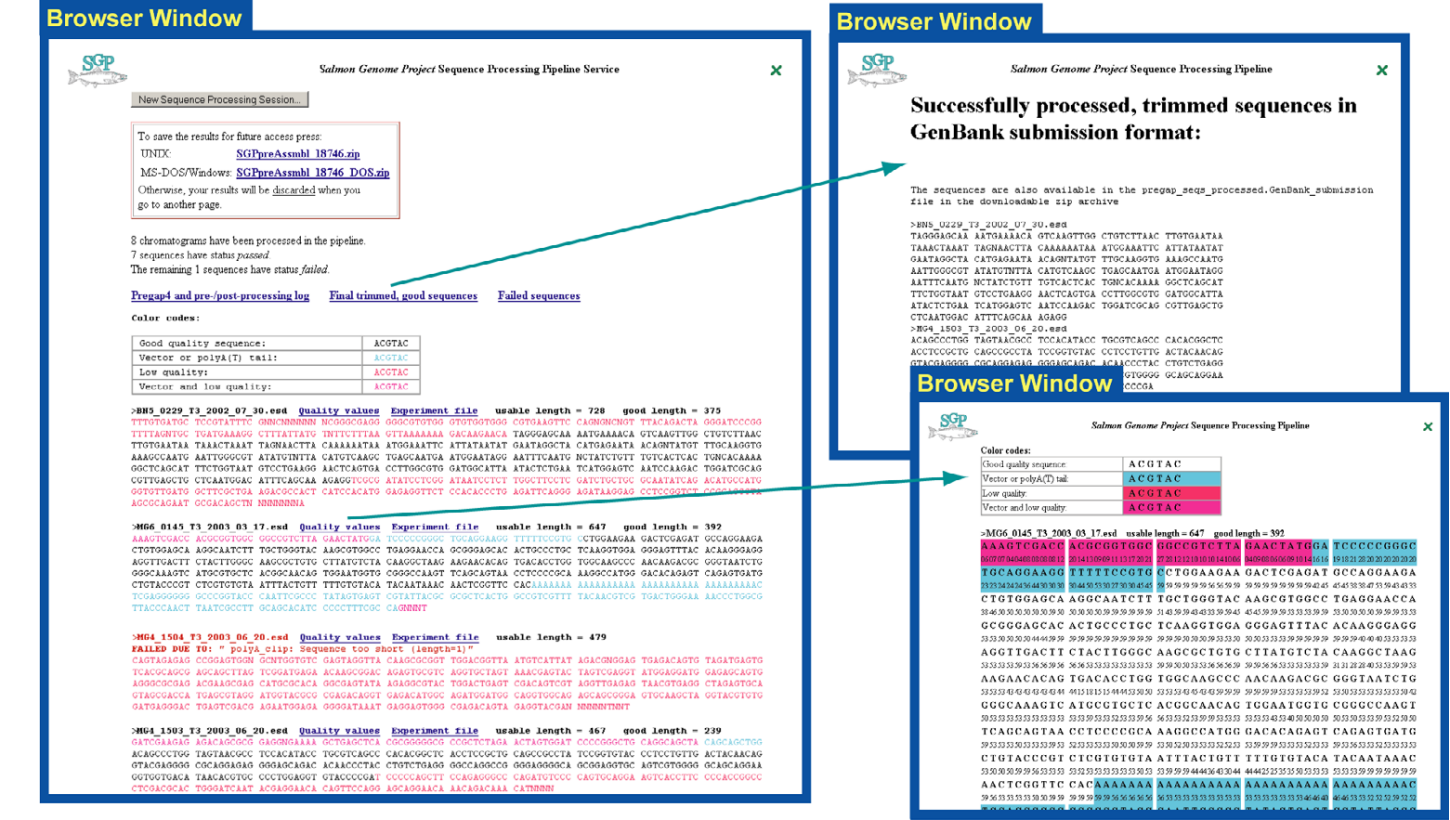
**preAssemble output**. Main HTML window of the preAssemble output and supplementary windows showing processed sequences in the Fasta format and detailed colour-coded processing results with Phred quality values, available for each sequence.

The main preAssemble html output file contains colour-coded processed sequences and links to files with supplementary data, Figure 1. Colour coding reflects marking done at different processing stages and provides an easy way of assessing results. The main html output page contains a self-explanatory summary of the processing results.

The following html output results files are linked to the main page.

(1) Final trimmed, good sequences: all 'passed' sequences in Fasta format with vector and low quality regions removed.

(2) Failed sequences: all sequences rejected at different processing stages.

(3) Processing log: log returned by the programs run by preAssemble.

For each sequence two additional files are available.

(1) Quality values: colour-coded sequence with the quality values assigned by Phred.

(2) Experiment file: Staden EXP file containing the detailed processing results.

The output also contains the following data: source trace files converted to the SCF format and the final EXP sequence files in separate directories, sequences in the Fasta format and sequences in the format compatible with the NCBI Sequin Gen Bank submission tool.

### Using preAssemble

Information on installing and running preAssemble as well as the full list and description of the output files is given in Help accessible on the preAssemble page at the SGP web site and distributed with preAssemble.

preAssemble is mainly intended for the Expressed Sequence Tags (EST) sequencing projects although other data can be processed. The pipeline processes chromatograms (trace files) produced by DNA sequencer machines and outputs results as the Staden Package [3,4] EXP format files which can be used for further processing, and also as web documents easily viewed with a browser. preAssemble is a relatively simple but powerful tool which can be used when there are just a few trace files or a substantial volume of data. The preAssemble web server can be most efficiently used for testing data quality for larger sequencing projects for which sample datasets can be processed, or for small scale data processing. For large scale production processing the preAssemble pipeline can be downloaded and installed locally.

preAssemble is written in Perl and runs under UNIX and LINUX. It is easy to install and use and was designed to perform automatic data processing and create output allowing detailed examination of the results. The pipeline performs pre-assembly processing or pre-processing, which is the first stage of data processing in a sequencing project if it is roughly divided into three parts – pre-processing, assembly and finishing, annotation. This approach was adopted since pre-processing results provide useful data for the projects where data processing steps differ from the standard routine such as outlined above or implemented in the Phred/Phrap/Autofinish package [1,2,6]. Additionally, examining the pre-processing results can help to revise the source data set, which can then be more efficiently processed using Phred/Phrap or similar software. Separate pre-processing can be used also when contigs assembly is not required and the output data will be analysed or further processed in a different way. Since the preAssemble pipeline produces Staden EXP files which are compatible with all modules of the Staden Package including the assembly module Gap4, this data can be used for subsequent assembly using Staden.

We did not use a relational database as part of this distribution of preAssemble to manage the data since in our view there is no need for this considering the number of operations and data complexity. In this respect preAssemble is different from for example the ESTWeb package [7]. There are less software requirements in comparison with ESTWeb, i.e. no relational database system to install and run, and no requirement for a web server installed on the user's computer. We tried to design preAssemble in such a way that a user with basic computer literacy can be comfortable with installing and running it. preAssemble will run with good results with the default parameters but all relevant parameter files are accessible and can be modified.

## Conclusion

preAssemble is a tool developed to perform pre-assembly processing of the DNA sequencing data on several sequencing quality parameters for small to large scale datasets, utilising standard Phred and Staden software. Two sets of results are produced: easy to read, self-explanatory but detailed html files designed to be viewed by a user, and data which can be used for further processing. The main application of preAssemble is to provide enhanced information allowing to assess sequencing data quality prior to further processing. This can be done by submitting jobs on the preAssemble web server for small datasets, or by downloading, installing and running a local version of preAssemble. Detailed instructions on how to use preAssemble are provided on the preAssemble web page.

## Availability and requirements

**Project name: **Salmon Genome Project preAssemble

Project home page:

Web access:  > SGP workbench > preAssemble

Download:  > Supplemental > Download preAssemble

**Operating system(s): **web access: not applicable, stand-alone: UNIX/LINUX

**Programming language: **Perl

**Other requirements: **web access: none, stand-alone: Perl 5.6, Phred, Staden package

**License: **SGP preAssemble license, free for academic and non-academic users

**Any restrictions to use by non-academics: **none

## Authors' contributions

AA conceived the study, coordinated and participated in software design and drafted the manuscript. JL worked on software design, carried out development and implementation, and participated in drafting the manuscript. AV worked on the software and web design and development.
